# Inhibition of midbrain cholinergic neurons impairs decision-making strategies during reversal learning

**DOI:** 10.3389/fnmol.2024.1481956

**Published:** 2024-11-21

**Authors:** Yuwoong Kim, Nadine K. Gut, Michael W. Shiflett, Juan Mena-Segovia

**Affiliations:** ^1^Center for Molecular and Behavioral Neuroscience, Rutgers University, Newark, NJ, United States; ^2^Department of Psychology, Rutgers University, Newark, NJ, United States

**Keywords:** pedunculopontine, cholinergic neurons, chemogenetic inhibition, reversal learning, behavioral flexibility, acetylcholine

## Abstract

**Introduction:**

The pedunculopontine nucleus (PPN) plays a role in coordinating complex behaviors and adapting to changing environmental conditions. The specific role of cholinergic neurons in PPN function is not well understood, but their ascending connectivity with basal ganglia and thalamus suggests involvement in adaptive functions.

**Methods:**

We used a chemogenetic approach in ChAT::Cre rats to explore the specific contribution of PPN cholinergic neurons to behavioral flexibility, focusing on the adaptation to shifting reward contingencies in a Reversal Learning Task. Rats were first trained in a non-probabilistic reversal learning task, followed by a probabilistic phase to challenge their adaptive strategies under varying reward conditions.

**Results:**

Motor functions were evaluated to confirm that behavioral observations were not confounded by motor deficits. We found that inhibition of PPN cholinergic neurons did not affect performance in the non-probabilistic condition but significantly altered the rats’ ability to adapt to the probabilistic condition. Under chemogenetic inhibition, the rats showed a marked deficiency in utilizing previous trial outcomes for decision-making and an increased sensitivity to negative outcomes. Logistic regression and Q-learning models revealed that suppression of PPN cholinergic activity impaired the adaptation of decision-making strategies.

**Discussion:**

Our results highlight the role of PPN cholinergic neurons in dynamically updating action-outcome expectations and adapting to new contingencies. The observed impairments in decision-making under PPN cholinergic inhibition align with cognitive deficits associated with cholinergic dysfunction in neurodegenerative disorders. These findings suggest that cholinergic neurons in the PPN are essential for maximizing rewards through the flexible updating of behavioral strategies.

## Introduction

Behavioral flexibility refers to the ability to adjust behavior in response to contingency changes in the environment (Lea et al., 2020; Scott, 1962). This process involves identifying contextual deviations from predicted outcomes, updating action-outcome contingencies, and inhibiting outdated behavioral strategies (Dajani and Uddin, 2015; Nilsson et al., 2015). These functions are distributed across several brain areas and encoded in specialized circuits. The action of neuromodulators, through their widespread long-range connectivity, has been suggested to play a central role in complex behavioral processes by coordinating neuronal activity across spatially distant circuits (see Harris-Warrick and Johnson, 2010 for a review). One such neuromodulator associated with behavioral flexibility is acetylcholine. Clinical evidence has established a correlation between behavioral rigidity and cholinergic depletion in neurodegenerative disorders such as Parkinson’s disease (PD; Müller et al., 2013), progressive supranuclear palsy (PSP; Warren et al., 2005) and multiple system atrophy (MSA; Benarroch et al., 2007), suggesting a key role in adaptive behavior.

The role of acetylcholine in flexible behavior is well documented (see Prado et al., 2017 for a review). For example, rats with enhanced cholinergic transmission using the cholinesterase inhibitor galantamine needed fewer trials to reach criteria in an attentional set-shifting task, indicating improved performance in behavioral flexibility (Nikiforuk et al., 2015). Galantamine also attenuated behavioral deficits induced by kynurenic acid exposure in rats during early development (Alexander et al., 2013). Age-related reversal learning deficits were alleviated with systemic administration of the acetylcholinesterase inhibitor tacrine (Tait et al., 2013). Likewise, muscarinic agonist administration significantly improved adaptation under changing contexts in a cross-maze (Ragozzino et al., 2012). In contrast, knockout of the acetylcholine transporter gene in mice triggered learning deficits during acquisition of a paired-associates learning task (Al-Onaizi et al., 2017) and in the Morris water maze (Kolisnyk et al., 2013). The impact of cholinergic transmission on behavioral flexibility has been shown to depend on the difficulty of the task, particularly those requiring sustained attention (Nikiforuk et al., 2015; Young et al., 2007). Thus, while the role of acetylcholine in tasks requiring behavioral adaptation has been established, the neuronal circuits underlying these functions have not been fully elucidated.

The pedunculopontine nucleus (PPN), a midbrain structure containing cholinergic neurons densely connected to the basal ganglia (Dautan et al., 2014; Martinez-Gonzalez et al., 2014) and the thalamus (Huerta-Ocampo et al., 2020), is implicated in the modulation of adaptive behavior, though the role of its cholinergic neurons remains unclear. Lesioning the entire PPN, including glutamatergic and GABAergic neurons, reduced rats’ accuracy in radial maze performance when new choices were introduced (Taylor et al., 2004), impaired learning of novel complex schedules of reinforcement (Wilson et al., 2009), and disrupted reversal learning in a spatial discrimination test when the previously baited arms were reversed after the acquisition phase (Syed et al., 2016). Furthermore, muscimol injections in the PPN impaired contingency updating and goal-directed behavior (MacLaren et al., 2013), suggesting an integrative role during behavioral performance, possibly by relaying sensorimotor and associative signals to midbrain dopaminergic neurons of the substantia nigra (Hong and Hikosaka, 2014). Likewise, *in vivo* calcium imaging showed increased activity of identified PPN cholinergic neurons during reward delivery in an attentional set-shifting task, and during error trials in a reversal learning task, suggesting their sensitivity to rule switches (Ruan et al., 2022). In contrast, PPN manipulations had no impact on the performance of previously learned contingencies (Alderson et al., 2004; MacLaren et al., 2013). These studies suggest that cholinergic neurons may participate in PPN functions associated with updating action-outcome contingencies (Mena-Segovia and Bolam, 2017), but further research is needed.

Here we tested the necessity of PPN cholinergic neurons in behavioral flexibility, specifically in adapting to changing action-outcome contingencies. This adaptation requires different strategies to maximize reward retrieval under conditions of certainty (non-probabilistic) or uncertainty (probabilistic). Using a chemogenetic approach in transgenic ChAT-Cre rats, we selectively and transiently suppressed PPN cholinergic activity during the execution of an operant non-probabilistic Reversal-Learning Task followed by a switch to a probabilistic task to assess changes in strategy that the rats use to maximize positive outcome. Chemogenetic inhibition did not affect performance during non-probabilistic reversal learning, but when reward probabilities changed, inhibition prevented the rats from adapting to the uncertainty of reward delivery. This disruption was due to reduced use of reward history and altered response to negative outcomes to guiding choices. Our results support a role of PPN cholinergic neurons in updating behavioral strategies when outcomes are uncertain.

## Methods

### Animals

In all experiments, heterozygous male (350-450 g) and female (220-260 g) ChAT::Cre + Long-Evans rats were used as experimental animals, where Cre recombinase was expressed under the choline acetyltransferase (ChAT) promoter (*N* = 6). ChAT::Cre-negative littermates were used as control (*N* = 6). Animals were food restricted to motivate tasks learning, maintaining their body weight at about 80 to 85% of *ad-libitum* levels. The housing was maintained on a 12-h light/dark cycle (lights on at 7:00 am), and behavioral tests were conducted around 03:00 PM. All experimenters were trained in handling rats to minimize stress during the experiments. All procedures were designed to minimize discomfort and were approved by the Institutional Animal Care and Use Committee at Rutgers University.

### Stereotaxic surgery

All surgical tools were sterilized before each procedure to minimize the risk of infection. Surgeries were conducted under deep isoflurane anesthesia (3–4% induction, 1–2% maintenance). Rats were placed on a temperature-controlled heating pad during surgery to prevent hypothermia. To selectively transduce inhibitory DREADDs (Designer Receptor Exclusively Activated by Designer Drug; hM4Di) in PPN cholinergic neurons, we injected 400 nL of AAV2-hSyn-DIO-hM4Di-mCherry (Addgene #44363) bilaterally into the PPN [from Bregma: (AP: −7.8 mm, ML: −1.8 mm, DV: −7.2 mm)] using a 1 μL syringe (SGE Analytical Science) connected to a power-assisted pump (UMC4, World Precision Instruments) at a rate of 40 nL/min. After the injection, the syringe tip was left in place for 15 min before withdrawal. Following surgery, rats received analgesics (buprenorphine, Ethiqa XR, 0.5 mL/kg, i.p.), and were monitored for three days.

### Histology

To verify DREADDs expression in cholinergic neurons in the PPN, we detected mCherry, a red fluorescent protein co-expressed with hM4Di. After the experiments, rats were deeply anesthetized via intraperitoneal injection (IP) (Euthasol,Virbac, 0.5 mL/kg) and transcardially perfused with phosphate buffer saline (PBS; pH 7.4), followed by ~200 mL of 4% paraformaldehyde (PFA) in 0.1 M phosphate buffer (PB; pH 7.4). Brains were extracted and post-fixed for 24 h in 4% PFA. Post-fixed brains were transferred to PBS containing sodium azide (PBS-azide, Sigma-Aldrich) and sectioned at 50-μm using a vibratome (Leica VT1200S). Sections were then incubated with antibodies against mCherry (rabbit; ab167453, Abcam, 1:500), and ChAT (goat; AB144P, Milipore, 1:500) or nNOS (neuronal nitric oxide synthase; goat; ab1376, Abcam, 1:1000). primary antibodies followed by fluorescent secondary antibodies (Cy5-anti-goat, 488-anti-rabbit, bothJackson ImmunoResearch Lab, 1:500). Sections were then examined on a confocal (FV-2000, Olympus) or fluorescence microscope (BZ-X800, Keyence). Brightness and contrast of the images were adjusted in Photoshop (Adobe Systems) or ImageJ (Fiji) for analysis.

### Open field test

Rats were tested in an open field (82 cm × 82 cm) for 25 min and tracked using ANY-maze software (Stoelting Co.). Prior to testing, animals were habituated to the field. Each rat was tested after receiving either clozapine N-oxide (CNO, agonist for inhibitory chemogenetic receptor—hM4Di; 1 mg/mL in 0.5% DMSO) or saline injections (IP), with the test order counterbalanced. Behavioral tests were conducted 40 min post-injection. Measured parameters included maximum speed and average speed (reported here), number of entries into the central zone, and total time spent in the central zone (not shown here). Data from the first five minutes were excluded from the analysis.

### Elevated ladder test

A 90 cm ladder with 21 rungs spaced 3 to 9 cm apart was placed horizontally, 30 cm above the ground and tilted at a 9.6-degree angle. To motivate the rats to cross, their home cage was placed at the far end. Each trial began when the rat was released at the start and ended upon entering the home cage. The test consisted of 3 trials, conducted after CNO or saline injections. The primary measure was rear paw slips, a known deficit following lesions of PPN cholinergic neurons (MacLaren et al., 2016).

### Instrumental reversal learning task

Animals were trained to perform an instrumental Reversal Learning Task [adapted from Parker et al. (2016)] in operant chambers (Med Associates). The task began with a center light above the food magazine. If the rat interrupted the beam in the magazine for more than 200 ms within 5 s, the right and left levers extended. The rat then chose a lever, resulting in either a rewarded trial (delivery of a 45-mg chocolate pellet (Bio-Serv) paired with a 0.5 s 6 kHz tone at 80 dB) or a non-rewarded trial (0.5 s white noise without reinforcement). Failure to interrupt the light beam for more than 200 ms within the 5 s time window resulted in a time-out. There was a 5 s interval between trials. The rewarded lever alternated between right and left in each session and switched every 10–13 correct responses, randomized within each block. Session consisted of 150 trials or 60 min, whatever came first.

In the non-probabilistic phase of the Reversal Learning Task, the food pellet was delivered with 100% probability after pressing the correct lever and 0% probability of reward after pressing the incorrect lever. In the probabilistic phase, the food pellet was delivered with a 70% probability after pressing the high-probability lever and 10% probability after pressing the low-probability lever. Rats were considered to have reached the learning criterion after meeting the following conditions: (1) A binomial cumulative distribution function (using MATLAB’s ‘binocdf’ function) at a chance level of 50% was used to determine if the rat’s choices were random, by comparing its actual performance to what would be expected if it were selecting randomly.; (2) a Minimum of 65% correct responses; and (3) The regression coefficient for a trial that was rewarded −1 trial back was above 1.5, indicating that the reward from the previous trial significantly influenced the rat’s current choice, consistent with the findings of Parker et al. (2016). Ten weeks after viral infusion, the rats’ performance was assessed following injection of CNO (1 mg/mL in 0.5% DMSO, IP) or vehicle (0.9% NaCl, IP). Behavioral tests were conducted 40 min post-injection. In the non-probabilistic phase, rats underwent 10 alternating sessions (5 NaCl, 5 CNO). After the completion of the non-probabilistic phase, the rats were trained in the probabilistic reversal learning paradigm. For testing, both groups initially received 4 sessions with saline to confirm learning similarities, followed by 4 sessions with CNO.

### Data analysis and statistics

**Figure 1:**
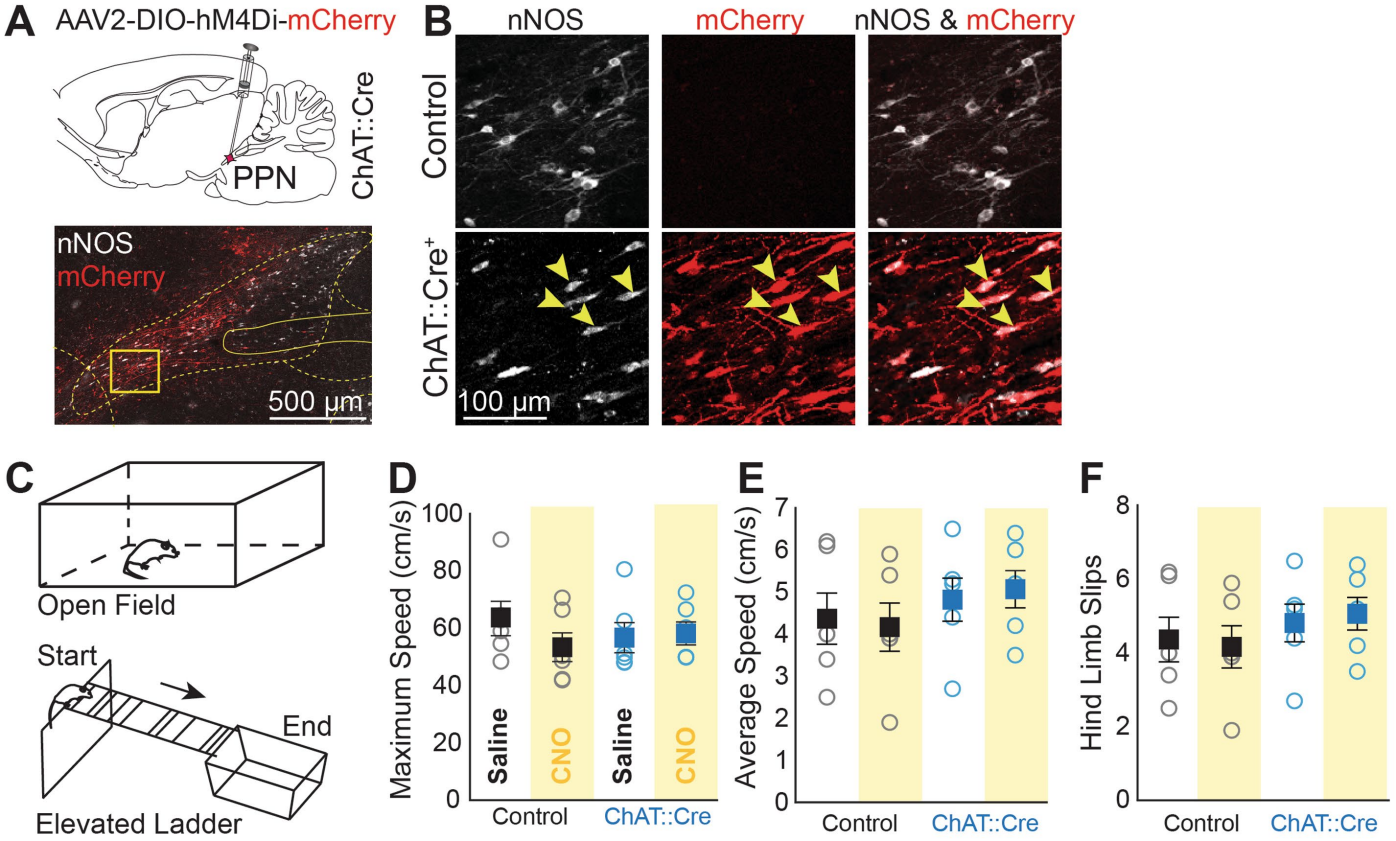
Chemogenetic suppression of PPN cholinergic neurons does not affect motor functions. **(A)** Schematic of bilateral virus injections of AAV2-DIO-hM4Di-mCherry in the PPN of ChAT::Cre + rats. Transduced neurons (red, mCherry/hM4Di) were confined to the PPN. **(B)** mCherry/hM4Di was selectively expressed in cholinergic neurons (arrows), which were labeled with antibodies against neuronal nitric oxide synthase (nNOS, white). No mCherry/hM4Di expression was observed in control animals. **(C)** Rats were tested in the Open Field and on an Elevated Ladder. **(D,E)** No differences in maximum speed and average speed were observed in experimental animals between saline and CNO administration, or when compared to control animals (group/drug interaction; max speed: Mixed ANOVA, F1,10 = 5.873, *p* = 0.036; *Post Hoc* Tests Bonferroni corrected: all comparisons *p* > 0.05; average speed: Mixed ANOVA, F1,10 = 0.849, *p* = 0.379). **(F)** In the Elevated Ladder Test, rats crossed a ladder with irregularly spaced rungs (3 to 9 cm intervals) set at a 9.6° downhill angle to measure motor coordination. CNO had no effect on hind limb slips (Mixed ANOVA, F1,10 = 0.595, *p* = 0.458) and no differences were observed in front paw slips, time to across, or speed (data not shown).

**Figure 2:**
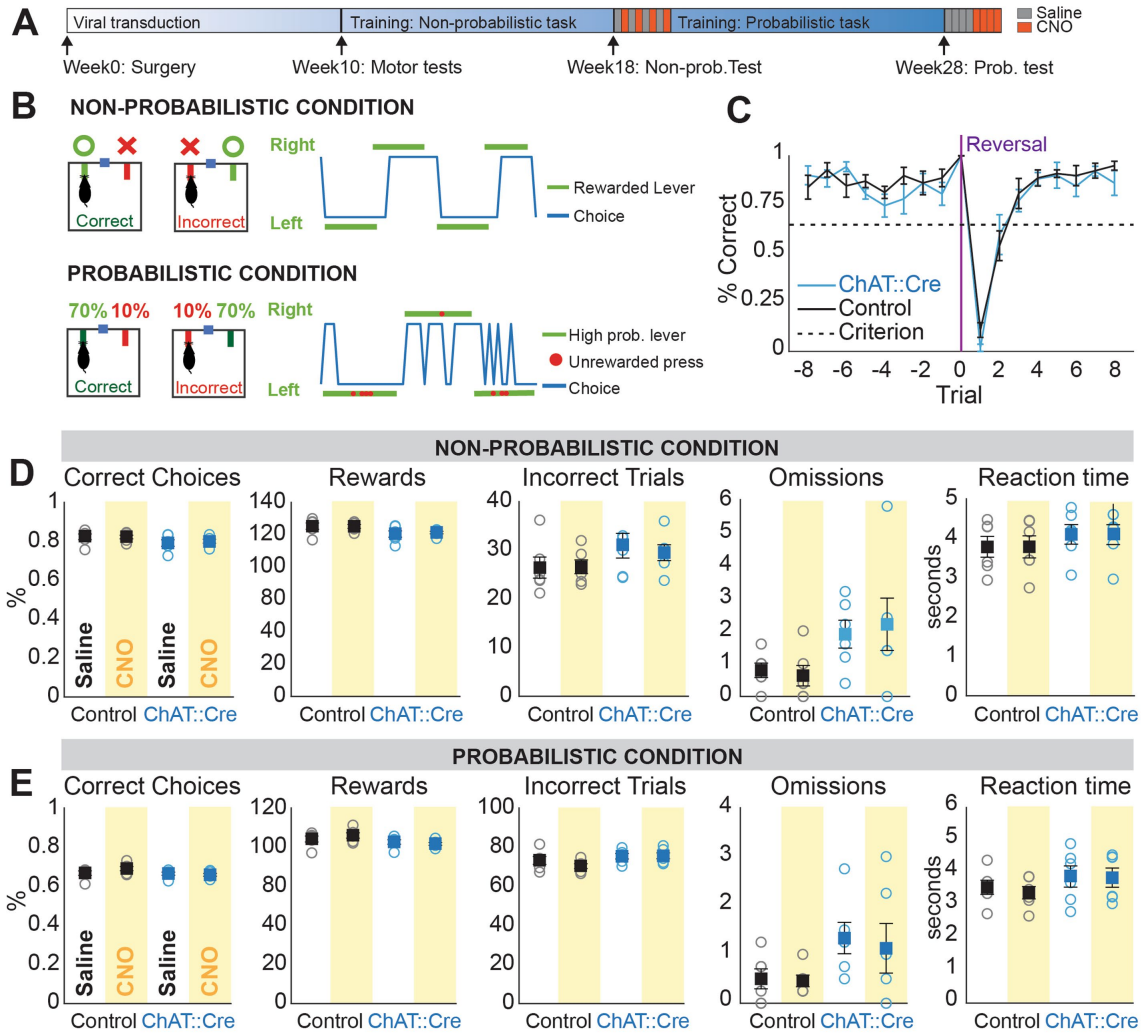
CNO administration does not affect performance in non-probabilistic and probabilistic reversal learning. **(A)** Experimental timeline. **(B)** Schematic of the behavioral paradigm: in the non-probabilistic condition, correct responses led to 100% reward delivery, while in the probabilistic condition, one lever delivered rewards 70% of the time and the other lever 10% of the time. Representative choice plots showing successful performance during non-probabilistic reversal learning (blue represents actual choices, green indicates the rewarded lever in each block) and probabilistic reversal learning (green represents high probability blocks and red dots indicate unrewarded choices during those blocks). **(C)** Probability of correct choices before reaching behavioral criterion, aligned to trial 0 (switching of the rewarded lever) on the last day of training administration. Both groups performance surpassed criterion and did not differ (Mixed ANOVA, F16,160 = 0.896, *p* = 0.575). **(D)** Non-probabilistic condition: No significant interaction between group (ChAT::Cre rats and controls) and condition (saline or CNO administration) was observed for percentage of correct responses (Mixed ANOVA, F1,10 = 0.435, *p* = 0.524), number of rewards (Mixed ANOVA, F1,10 = 0.277, *p* = 0.610), number of incorrect trials (Mixed ANOVA, F1,10 = 0.462, *p* = 0.512), number of omissions (Mixed ANOVA, F1,10 = 0.799, *p* = 0.392), or reaction time (Mixed ANOVA, F1,10 = 0.001, *p* = 0.971). **(E)** Probabilistic condition: No differences were observed for the same comparisons in percentage of correct responses taking into account testing order (“sessions”) (Linear Mixed Models (LLM), fixed effects: group, condition, session, F1,10.32 = 1.161, *p* = 0.306), number of rewards (LLM, F1,10.06 = 0.788, *p* = 0.395), number of incorrect trials (LLM, ANOVA, F1,10 = 0.732, *p* = 0.412), number of omission (LLM, F1,11.19 = 0.062, *p* = 0.807), and reaction time (LLM, F1,10.17 = 0.143, *p* = 0.713).

**Figure 3:**
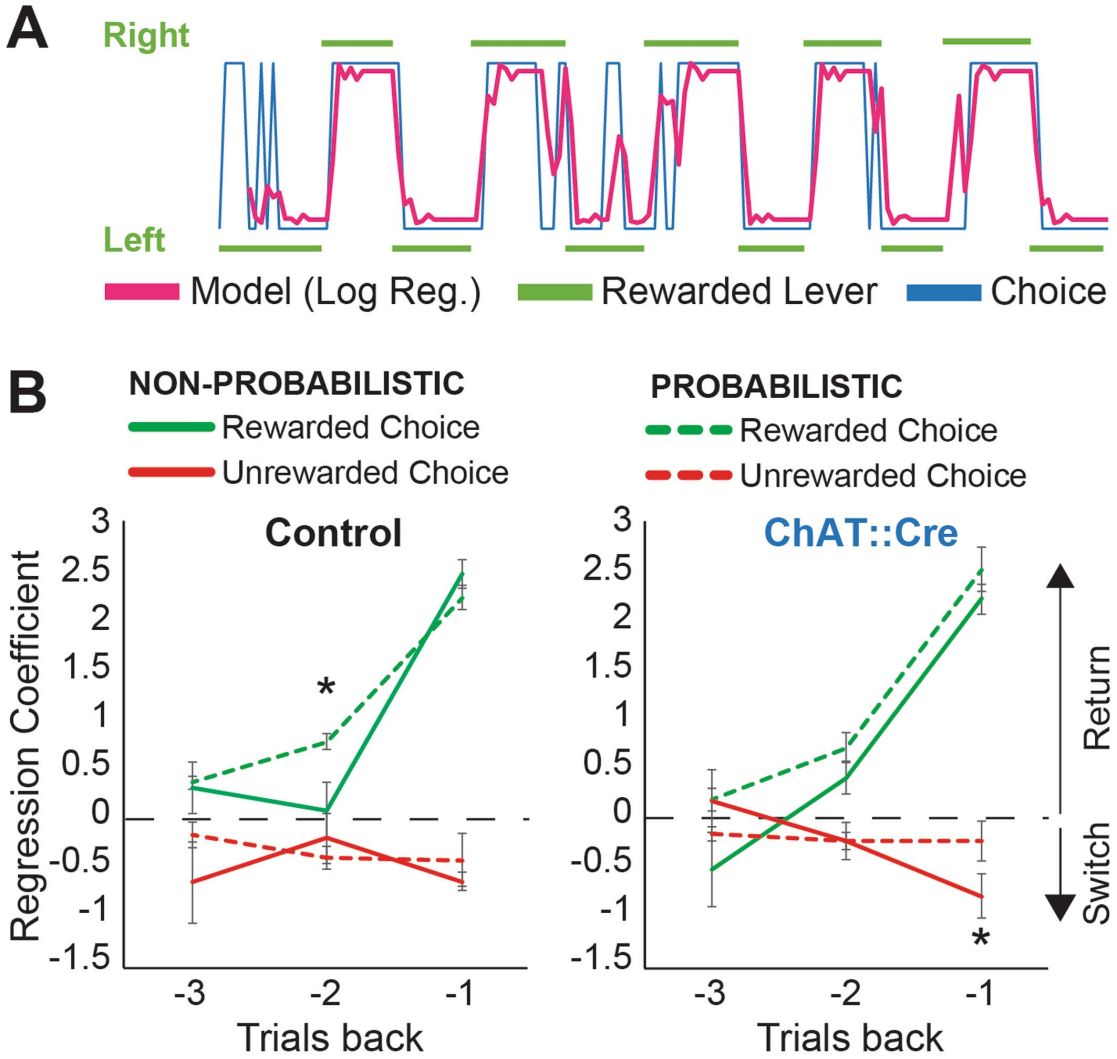
Logistic regression indicates different adaptation patterns to probabilistic reversal learning. **(A)** Model predictions show that the logistic regression model fits real data (red: the model prediction, blue: actual choices, green: rewarded levers). **(B)** Regression coefficient values from the logistic regression, with green and red lines indicating the reward predictors and non-reward predictors, respectively. Higher regression coefficients indicate a greater likelihood of selecting the previously rewarded lever in the current trial. Rewards from −2 trials back had significantly more weight on the choices of control animals after introducing the probabilistic condition (one-tailed paired sample *t*-test, −2 trials back; reward predictor, non-probabilistic vs. probabilistic reversal learning, *t*(5) = 2.532, *p* = 0.026). Negative feedback (no reward) from −1 trial back had significantly less influence on the choices of experimental animals in the probabilistic condition (one-tailed paired sample *t*-test, −1 trial back; non-reward predictor, non-probabilistic vs. probabilistic reversal learning, *t*(5) = 2.246, *p* = 0.037).

**Figure 4:**
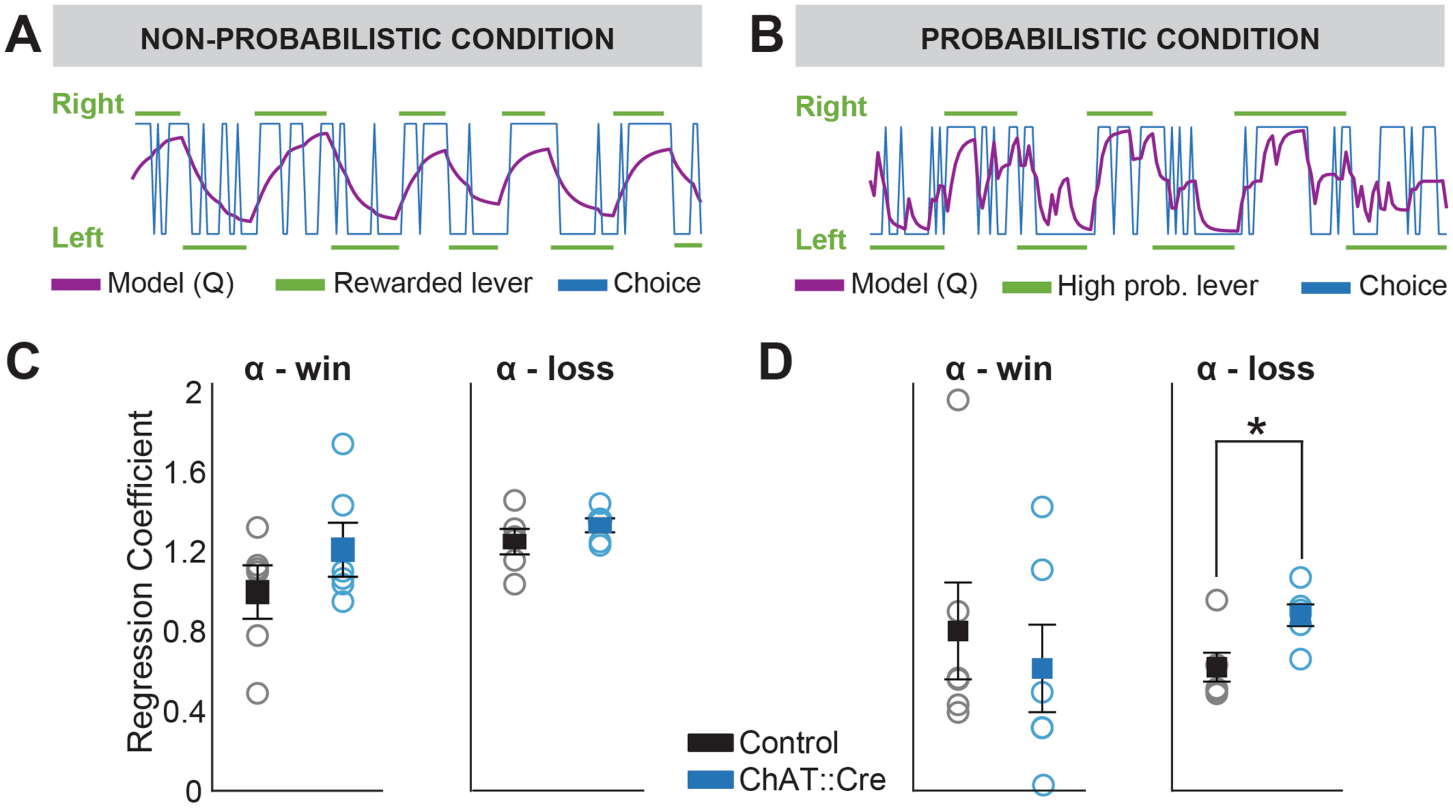
Q-learning model reveals increased sensitivity to negative feedback in experimental animals during probabilistic reversal learning. **(A,B)** Q-learning model estimates the update of action-value (Q-values) based on previous rewards and reward omissions. Blue and green lines represent actual choices and rewarded levers (high probability levers in case of the probabilistic condition), respectively. The purple line represents the model’s prediction of the animal’s choice. **(C)** No significant differences were found between groups for α*_win_* (the degree to which previous rewards guided choices) or α_loss_ (the degree to which negative feedback influenced choices) under CNO administration during the non-probabilistic condition (one-way ANOVA, α*_win_*: F1,10 = 0.291, *p* = 0.579 and α*_loss_*: F1,10 = 1.257, *p* = 0.288). **(D)** While α*_win_* did not significantly differ between groups under CNO in the probabilistic condition (one-way ANOVA, F1,10 = 0.329, *p* = 0.579), there was a significant difference in α*_loss_* (one-way ANOVA, F1,10 = 8.284, *p* = 0.016), indicating a greater influence of reward omission in experimental animals under CNO compared to controls. No significant effects or interactions were observed with saline administration (data not shown).

Med-PC data files were exported and post-processed using custom MATLAB scripts. JASP software (University of Amsterdam) was used to calculate mixed ANOVAs (Figures 1, 2C,D; between factor = group [control vs. ChAT::Cre], within factor = condition [saline vs. CNO]), Linear Mixed Models (Figure 2E) to account for learning effects over sessions (fixed factors: group, condition, session number; random effect: individual subjects). Paired-sample *t*-test (Figure 3) and univariate ANOVAs (Figure 4; control vs. ChAT::Cre) were also used.

A logistic regression model (Parker et al., 2016) was implemented in a custom Matlab code using the ‘glmfit’ function. The logistic regression equation is expressed as follows:


$$\begin{array}{c} \log\left(\frac{\text{choice}\left(\text{now}\right)}{1-\text{choice}\;\left(\text{now}\right)}\right)=\beta_{0}+\sum_{j=0}^{\text{trialBack}}\beta_{j}^{Rew}Rew\left(\text{now}-j\right)\\ +\sum_{j=0}^{\text{trialBack}}\beta_{j}^{\text{nRew}}\text{nRew}\left(\text{now}-j\right) \end{array}$$

where *Choice (now)* represents the probability of choosing the correct lever on the current trial. *Rew* is a reward predictor variable, with +1 for a rewarded right press, −1 for a rewarded left press, and 0 for no reward. *nRew* is a non-reward predictor, with +1 for an unrewarded right press, −1 for and unrewarded left press, and 0 for a reward. *j* is the number of previous trials. The coefficients *β_0_, β_Rew_*, and *β_nRew_* represent the strength of the relationship between outcomes and current choices averaged across animals (Bates et al., 2015; Parker et al., 2016). Model fit was assessed using log-likelihood against a chance model (0.5 probability per choice).

To model the learning strategies used by rats in each contingency, we applied the Rescorla-Wagner model (Q-learning; Verharen et al., 2018) which is based on their choice behavior.


$$RPE_{t-1}=\{\begin{array}{c} 1-Q_{t-1}\;\left(\text{win}\;\text{trials}\right)\\ 0-Q_{t-1}\;\left(\text{loss trials}\right) \end{array}$$

The value *Q*, represents the rats’ estimate of the value of a specific action at a given time, *t*. This value is updated based on the rat’s previous experiences and expectations. The calculation of *Q* at time *t* is based on the previous value, *Q_t-1_*, and the learned association between the action and its outcome. This learned association is influenced by the reward prediction error (RPE), which is the difference between the expected outcome and the actual outcome.


$$Q_{t}=\{\begin{array}{c} Q_{t-1}+a_{\text{win}}\;\cdot RPE_{t-1}\;\left(\text{win}\;\text{trials}\right)\\ Q_{t-1}+a_{\text{loss}}\cdot RPE_{t-1}\;\left(\text{loss trials}\right) \end{array}$$

Two learning rate parameters, *α_win_* and *α_loss_*, describe how the rats adjust their expectations based on positive (win or rewarded trials) and negative (loss or non-rewarded trials) RPE, respectively. These learning rates are determined by the outcomes of the rats’ prior experiences and expectations, using RPE to adjust the learning process. The learning rates *α_win_* and *α_loss_* determine how much the animals learn from positive and negative outcomes, respectively.


$$P_{t}=\frac{e^{\beta \;\cdot \;Q_{t}}}{\left(1-e^{\beta \;\cdot \;Q_{t}}\right)+e^{\beta \;\cdot \;Q_{t}}}$$

The action outcome values, *Q_t_*, are transformed into action probabilities (*P*) of pressing each lever by using a softmax function, where *P_t_* represents the probability of choosing the right lever and *1-Pt* represents the chance of choosing the left lever in trial *t*. The parameter *β*, determines how much the rats’ actual choice is influenced by the lever values versus random selection.

The parameters *α_win_*, *α_loss_*, and *β* were determined by minimizing the negative likelihood of the rats’ actual choices using the ‘fmincon’ function in Matlab, computed per animal to avoid local minima.

## Results

### Chemogenetic suppression of PPN cholinergic neurons does not affect motor functions

The PPN, a key component of the mesencephalic locomotor region, is known to induce motor activity, but recent studies have challenged the role of cholinergic neurons in movement control. Before assessing the role of PPN cholinergic neurons in behavioral flexibility through chemogenetic inactivation, we first evaluated the general motor performance to ensure that the performance in the Reversal Learning Task was not influenced by motor inhibition. Following inhibitory DREADDs transduction (Figures 1A,B), ChAT::Cre + rats and controls were tested in the Open Field and the Elevated Ladder Test following saline or CNO injections (Figure 1C). The maximum speed and average speed in the Open Field did not differ between groups or conditions (Figures 1D,E). Motor coordination was also unaffected, as indicated by the number of hindlimb slips while traversing an elevated ladder with irregularly spaced rungs (Figure 1F). These results show that transient chemogenetic inactivation of PPN cholinergic neurons did not impair the overall motor function.

### Inhibition of PPN cholinergic neurons during non-probabilistic and probabilistic reversal learning

To test whether PPN cholinergic neurons modulate behavioral flexibility, we examined the same rats in an instrumental Reversal Learning Task during chemogenetic inactivation, using the strategy described above. Rats were trained in a self-paced, instrumental task (adapted from Parker et al., 2016; see Figure 2A for the experimental timeline) involving a choice between two levers (Figure 2B; see methods). They were then trained and tested in two conditions: a non-probabilistic version, where one lever always delivered a reward (100%) and the other lever did not (0%), and a probabilistic version, where one lever had a high probability of reward (70%) while the other a low probability (10%). After a minimum of 10 rewarded trials, contingencies were reversed, with randomized blocks of 10-13 trials. Testing began once all animals reached stable performance in the non-probabilistic task (Figure 2C; see methods for criteria).

We first evaluated several parameters, including the percentage of correct responses, numbers of rewards, incorrect trials, omissions, reaction time and reversals to assess performance in both the non-probabilistic (Figure 2B) and probabilistic condition (Figure 2D). None of these parameters were significantly different between groups or across trials in either condition (Figures 2D,E). Other parameters such as bias toward one lever, and percentage of correct responses at reversal, were also not significantly different. These data suggest that PPN cholinergic activity is not necessary to correctly perform a previously learned task under certain (i.e., non-probabilistic) or uncertain (i.e., probabilistic) conditions.

### Changes in decision making strategies following cholinergic inhibition

The PPN has been shown to be necessary for updating previously learned action-outcome associations, whereas acquisition itself is not affected by various manipulations of PPN functioning (Alderson et al., 2004; MacLaren et al., 2013). We therefore examined which strategy rats used to solve both the non-probabilistic and probabilistic versions of the task, and how they adapted their strategy from a condition with certain rewards to a strategy with uncertainty. We first examined how the animals used outcomes from previous trials to inform their choices. To predict the animals’ choices, we applied a logistic regression model based on previous choices (left or right lever) and outcomes (reward or no reward). Model predictions fit the animal’s choice (Figure 3A; pseudo-R^2^ non-probabilistic condition: 0.43 to 0.91, median 0.6673; probabilistic condition: 0.25 to 0.61, median 0.43). In the non-probabilistic task, where action-outcome contingencies are absolute, the reward from the previous trial (−1 trial back) is the most influential outcome that informs a choice. The logistic regression confirmed this for both groups (Figure 3B; one-way ANOVA, *reward predictor*, fixed factor: trial back, control animals: F2,15 = 30.706, *p* < 0.001; post-hoc Tukey correction, −1 vs. −2: *p* < 0.001; −1 vs. −3: *p* < 0.001 experimental animals: F2,15 = 39.743, *p* < 0.001; post-hoc Tukey correction, −1 vs. −2: *p* < 0.001; −1 vs. −3: *p* < 0.001). However, in the probabilistic task, animals need to consider their reward history. In control animals, rewarded choices from −2 trials back significantly increased the likelihood to return to the same lever, after switching to the probabilistic condition (Figure 3B; one-tailed paired sample *t*-test, −2 trials back; reward predictor, non-probabilistic vs. probabilistic reversal learning, *t*(5) = 2.532, *p* = 0.026). ChAT::Cre rats failed to make this adjustment (no significant difference between probabilistic and non-probabilistic reward predictor values at −2 back Figure 3B, *T*(5) = 1.241, *p* = 0.135). Additionally, the control group demonstrated a clearer separation between rewarded and unrewarded choices at the −3 trial, whereas ChAT::Cre rats did not utilize this information as effectively, though this difference was not significant. These data show that under PPN cholinergic inhibition, the integration of previous outcomes in decision-making was disrupted.

In contrast to logistic regression, which models the use of previous outcomes in a binary manner, the Q-Learning model (Verharen et al., 2018) integrates both immediate rewards and expected future rewards to iteratively improve the strategy. It continuously updates the learning rule, leading to more effective decision-making over time. It describes a value function that guides decisions based on the value of an action in a given state, the contingency between stimulus and reward, and the RPE. RPE is calculated as the difference between the old value and a new expected value informed by the sum of the immediate reward and the discounted expected value of the future action. We found that the Q-learning model fit the behavioral data (Figures 4A,B; log likelihood range non-probabilistic condition: −58.2 to −132.81; probabilistic condition: −88 to −54; mean log likelihood of chance model: −103.965). In the non-probabilistic condition, both groups updated action values similarly in response to positive outcomes (win) and negative outcomes (loss) during chemogenetic suppression (Figure 4C). However, in the probabilistic condition, negative outcomes (losses) had a stronger influence on action value updates in experimental animals compared to controls (Figure 4D; one-way ANOVA, α*_loss_*, control vs. experimental animals, F1,10 = 8.284, *p* = 0.016). This suggests that inhibition of PPN cholinergic neurons specifically affects how negative outcomes in conditions of uncertainty influences learning strategies.

## Discussion

In this study, we investigated the role of PPN cholinergic signaling in behavioral flexibility using a Reversal Learning Task combined with chemogenetic suppression of PPN cholinergic neurons. To ensure that motor impairments did not confound our results, we assessed general motor functions. Results from the Open Field and the Elevated Ladder Test indicated that general motor functions, including motor coordination, remained intact under chemogenetic suppression of PPN cholinergic neurons (Figure 1). The same rats were then trained in a Reversal Learning Task and subjected to two different conditions: a non-probabilistic task and a probabilistic task. Each task required distinct strategies for successful performance: in the non-probabilistic task, an unrewarded choice indicated a change in contingencies, prompting the rats to switch to the other lever. Conversely, in the probabilistic task, a reward omission on the high-probability lever did not necessarily signal a change in contingencies. To maximize reward acquisition, rats had to adjust their learning strategies and update their expected action-outcome association. There is substantial evidence supporting the role of the PPN in cognitive function, in particular during complex tasks that require shifts in learning strategies, such as contingency changes or extinction (Alderson et al., 2004; MacLaren et al., 2013; Wilson et al., 2009). Further, PPN cholinergic neurons directly influence structures critical for behavioral flexibility, including the parafascicular thalamic nucleus (Bradfield et al., 2013; Brown H. D. et al., 2010) and the striatum (Dautan et al., 2020).

The effects of PPN cholinergic inactivation on reversal learning in our task revealed subtle differences in performance strategies. In contrast, a study examining the effect of PPN lesions on probabilistic reversal learning in a 4-arm T-Maze found that acquisition of reversal learning was impaired, as indicated by an increased number of trials required to reach criterion due to an increase of regressive errors (Syed et al., 2016). Our study differed from Syed et al.’s work in that we tested the effect of PPN cholinergic inhibition *after* the rats had already reached the performance criterion. Silencing cholinergic neurons after reaching this criterion did not affect their performance (Figure 2). In contrast, Syed et al. demonstrated that PPN lesions not only affect overall performance, but also altered the learning strategy used to solve the probabilistic Reversal Learning Task. Consequently, we examined these learning strategies in our study.

We examined how rats integrated previous outcomes with current choices after transitioning from the non-probabilistic task to the probabilistic task. During the non-probabilistic task, rats relied primarily on a “win-stay/lose-shift” strategy, in which the rat repeated an action that in the previous trial resulted in a reward (reward ➔ return to same lever). Unrewarded choices and the reward history (2 or 3 trials prior) had minimal influence on the rats’ choice, as observed in both groups. However, when uncertainty was introduced, the rats had to place greater weight on the outcomes of previous choices (Figure 3). Control rats adapted their strategy accordingly, with rewarded choices from 2 trials back increasing the likelihood of staying on the same lever. In contrast, experimental rats lacking PPN cholinergic activity, did not adjust their strategy in the same way. Instead, they used a more myopic strategy that heavily weighted the most recent win trial.

For successful learning and adaptation, dopamine neurons of the midbrain provide a teaching signal that encodes outcome uncertainty based on reward prediction error (RPE). PPN cholinergic neurons directly modulate dopamine activity in the midbrain, encoding the predicted reward value and the actual value of the delivered reward, essential information for computing the RPE (Hong and Hikosaka, 2014; Norton et al., 2011; Okada and Kobayashi, 2013; Thompson and Felsen, 2013). Notably, a recent study demonstrated that PPN cholinergic neurons not only respond to reward, but also show increased activity in response to changes in stimulus-outcome contingencies following reward omission (Ruan et al., 2022). Ruan et al. observed an increase in perseverative errors and an increased time to reach criterion in the reversal learning phase of an attentional set-shifting task. Using calcium imaging with fiber photometry, they showed that PPN cholinergic neurons significantly increased their activity when a previously rewarded choice became unrewarded, even more so than in response to rewards. These neurons were particularly sensitive to outcomes that deviated from expectations during reversal learning (but not during non-reversal learning), suggesting that PPN cholinergic activity encodes information necessary for adjusting the expected value of an action. This activity is essential in the decision-making process about whether to modify behavior. Logistic regression does not model the learning process but instead determines the influence of previous outcomes in a binary manner, quantifying the probabilities of two possible choices: stay or switch. This approach does not account for the dynamics of long-term reward maximization and provides no insight into how the actions values are updated during task performance. Therefore, we used a Q-Learning model to understand how rats learned the value of their actions in specific states to maximize cumulative rewards. We found that during PPN cholinergic inhibition in the probabilistic task, negative feedback (reward omissions) had a greater influence on the rate at which the action-value function (Q-value) was updated compared to control animals (Figure 4). This can be interpreted as an enhanced sensitivity to negative feedback and a faster adaptation to unrewarded choices. Animals adjust their behavior more quickly when expected rewards are not received and abandon unsuccessful actions more rapidly. This seems to contradict previous findings of impaired reversal learning during PPN silencing. However, this also suggests a shift in decision-making balance, making the animals more risk-averse. They prioritize avoiding losses over balancing wins and losses. In a probabilistic learning task, where reward contingencies are uncertain, this is not a successful strategy. Negative feedback needs to be balanced with previous rewarded choices, as demonstrated by the logistic learning model in control animals, who considered rewards from earlier trials, not just the most recent ones. Our data reveals the involvement of PPN cholinergic neurons in adaptive decision-making under uncertainty. It expands our understanding of their role in reinforcement learning and behavioral flexibility, indicating a more nuanced role than previously assumed. The encoding of states that require behavioral change is context dependent, and PPN cholinergic activity is necessary for maintaining the balance between integrating of rewards and losses in adaptive learning.

PPN cholinergic cell loss occurs in certain types of parkinsonism, including Parkinson’s disease (PD), progressive supranuclear palsy (PSP) and multiple system atrophy (MSA). These neurodegenerative disorders are characterized by motor deficits, such as gait and balance impairments (Benarroch et al., 2007; Hirsch et al., 1987), and cognitive deficits, such as deficits in signal detection tasks (Kim et al., 2017), dementia, and perseverant responses (Brown R. G. et al., 2010). Cognitive deficits have been associated with reduced cholinergic transmission in the thalamus (Müller et al., 2013). Furthermore, thalamic acetylcholine-esterase (AChE) activity is lower in PSP than in PD patients, as measured by AChE positron emission tomography (Gilman et al., 2010), which correlates with a greater loss of cholinergic PPN neurons in PSP compared to PD. Given that approximately 95% of cholinergic transmission in the thalamus originates from the PPN (Bolton et al., 1993; Sofroniew et al., 1985), it is likely that the cognitive impairment observed in parkinsonism in at least partially explained by the loss of PPN cholinergic neurons. The cognitive symptoms associated with reduced cholinergic signaling in the thalamus align with our results, which show deficits in behavioral strategies under changing contingencies, further supporting the role of PPN cholinergic neurons in behavioral flexibility.

## Data Availability

The raw data supporting the conclusions of this article will be made available by the authors, without undue reservation.
